# An Intercellular Flow of Glutathione Regulated by Interleukin 6 Links Astrocytes and the Liver in the Pathophysiology of Amyotrophic Lateral Sclerosis

**DOI:** 10.3390/antiox10122007

**Published:** 2021-12-16

**Authors:** Rafael López-Blanch, Rosario Salvador-Palmer, José M. Estrela, Elena Obrador

**Affiliations:** 1 Department of Physiology, Faculty of Medicine and Odontology, University of Valencia, 46010 Valencia, Spain; loblanch@alumni.uv.es (R.L.-B.); rosario.salvador@uv.es (R.S.-P.); jose.m.estrela@uv.es (J.M.E.); 2 Department of Physiology, Faculty of Pharmacy, University of Valencia, 46010 Valencia, Spain

**Keywords:** amyotrophic lateral sclerosis, liver, astrocytes, motor neurons, mitochondria, glutathione, oxidative stress

## Abstract

Oxidative stress has been proposed as a major mechanism of damage to motor neurons associated with the progression of amyotrophic lateral sclerosis (ALS). Astrocytes are the most numerous glial cells in the central nervous system and, under physiological conditions, protect neurons from oxidative damage. However, it is uncertain how their reactive phenotype may affect motor neurons during ALS progression. In two different ALS mouse models (SOD1^G93A^ and FUS-R521C), we found that increased levels of proinflammatory interleukin 6 facilitate glutathione (GSH) release from the liver to blood circulation, which can reach the astrocytes and be channeled towards motor neurons as a mechanism of antioxidant protection. Nevertheless, although ALS progression is associated with an increase in GSH efflux from astrocytes, generation of reactive oxygen species also increases, suggesting that as the disease progresses, astrocyte-derived oxidative stress could be key to motor-neuron damage.

## 1. Introduction

Amyotrophic lateral sclerosis (or ALS) is a disease of the central nervous system (CNS) characterized by a progressive degeneration of motor neurons (MNs) in the cerebral cortex (upper MNs), brainstem and spinal cord (lower MNs) [1]. The consequence is muscle weakness, which can progress to paralysis, spreading from one body region to another [2]. Most cases (90–95%) have no genetic background (sporadic ALS, SALS), and their cause is unknown, whereas in 5–10% of cases, a specific genetic mutation (the most common are found in C9orf72, SOD1, FUS, or TARDBP) is the main underlying mechanism (familial ALS, FALS) [3]. In the pathophysiology of the disease, neuroinflammation and oxidative stress are main mechanisms leading to neurodegeneration and MN death [4].

Progression of the disease is associated with reactive glia- and immunity-dependent neuroinflammation [5]. As a consequence, an increase in levels of inflammation-related cytokines can be detected in the cerebrospinal fluid (CSF) and blood of murine models and ALS patients [6]. Interleukin 6 (IL6) is among these cytokines [6,7]. IL6 seems to have important function in the CNS, i.e., neurogenesis and response of neurons and glial cells following different types of injuries [8]. However, although it is difficult to evaluate inflammation of the CNS or the relationship between neuroinflammation and disease progression in ALS patients, it has been reported that the common IL6 receptor 358 Ala variant (IL6R358Ala) and trans-signaling are disease modifiers in ALS [7].

Glutathione (L-γ-glutamyl-L-cysteinyl-glycine; GSH), the most prevalent non-protein thiol in mammalian cells and a physiological antioxidant, is involved in many cellular functions [9]. Cys availability and γ-glutamyl-cysteine synthase activity are rate-limiting factors for the synthesis of GSH [10]. A decrease in antioxidant defense associated with alterations in GSH metabolism have been suggested as potentially negative factors involved in the progression of ALS [11]. Astrocytes are the most abundant type of macroglial cells in the CNS [12] and provide Cys and GSH to neurons [13]. It seems obvious that this function and the pro-inflammatory reactive response of astrocytes during ALS progression are in contradiction.

We found that IL6 plays a role in inducing the release of liver GSH in models of metastatic melanoma [14]. Hepatic GSH is considered the main systemic reservoir of this tripeptide and may be released to the blood and reach other organs and cells [15]. We explored whether this mechanism is also activated in ALS murine models, as well as its impact on GSH levels and generation of reactive oxygen species (ROS) in astrocytes.

## 2. Materials and Methods

### 2.1. Mice

Control (wild-type, WT) B6SJLF1/J mice https://www.jax.org/strain/100012 (accessed on 1 December 2020) (The Jackson Laboratory, Mount Desert Island, ME, USA). B6.Cg-Tg(SOD1*G93A)1Gur/J https://www.jax.org/strain/004435 (accessed on 1 December 2020) mice, which are hemizygous for the SOD1^G93A^ transgene with transgenic expression of a G93A mutant form of human SOD1. B6; SJL-Tg(Prnp-FUS*R521C)3313Ejh/J https://www.jax.org/strain/026406 (accessed on 1 December 2020) mice, where the majority of the transgenic FUS-R521C protein is nuclear.

### 2.2. Neurological Score

Neurological score in mice was determined weekly, by visual inspection, starting at approx. postnatal day 45. Neurological score is based on the scale of Weydt et al. [16]. Scores indicate the following: “0” a healthy mouse; “1” the presence of tremors in the hind legs; “2” difficulties in separating the hind legs when suspended by the tail; “3” difficulties walking; “4” unable to walk on all four legs, so access to food and water has to be facilitated. At a score of “5” (unable to right themselves), the animals are euthanized for ethical reasons. Onset is defined as the earliest time when mice show symptoms (score < 4) for ≥2 consecutive weeks.

### 2.3. Rotarod Test

This test is widely used to evaluate the neuromotor coordination of rodents. We used a touchscreen rotarod of Panlab/Harvard Apparatus (Cornellá de Llobregat, Spain). Each animal was given three trials, and the maximum period (seconds) that it could remain on a rotating axle (3.5 cm diameter; speed of rotation 15 rpm) without falling was recorded. The test was stopped after an arbitrary limit of 180 s. In the first 2 weeks, an adaptation period of three trials was performed before beginning the test recordings.

### 2.4. Isolation and Incubation of Hepatocytes

We followed the Berry and Friend method [17]. Hepatocytes were purified from the crude cell suspension by density gradient centrifugation in a vertical rotor [18]. The crude suspension (50 mg dry wt in 2 mL) was added to a medium (40 mL) containing 40% (*v*/*v*) of Percoll, 3% (*w*/*v*) of defatted bovine serum albumin, 10% DMEM, 10 mM MOPS, 120 mM NaCl, and 6.7 mM KCl, 1.2 mM CaCl_2_ and adjusted to pH 7.4 with 0.1 N NaOH. Centrifugation was performed at 4 °C in a Beckman-Coulter Optima XL-100K (7 × 10^4^ gav for 15 min). Metabolic viability and integrity of isolated hepatocytes was assayed as previously described [18]. For incubations in Erlenmeyer flasks, hepatocytes (10–12 mg dry wt/mL) were suspended at 37 °C in KHBM (pH 7.4) containing 1.3 mM CaCl_2_. The gas atmosphere was 95% O_2_–5% CO_2_.

### 2.5. Glutathione and Glutathione Disulfide Levels

Glutathione (GSH) and glutathione disulfide (GSSG) were measured by liquid chromatography–mass spectrometry (LC/MS) as previously described [19]. Cell processing followed the published methodology, where rapid N-ethylmaleimide derivatization was used to prevent GSH auto-oxidation [20].

### 2.6. Cerebrospinal, Blood and Liver Sample Collection

Whole-blood samples (0.5 mL) were obtained from the saphenous vein, collected in standard red-topped Becton Dickinson (Franklin Lakes, NJ, USA) tubes and allowed to clot for 30 min. Samples were centrifuged at 1500× *g* × 10 min (4 °C). Supernatants were stored at −80 °C. Samples of CSF were obtained as described in detail by Lim et al. [21]. Liver samples were quickly dissected, washed at 4 °C in KHBM (pH 7.4) without Ca^2+^ or Mg^2+^ and containing 0.5 mM EGTA, dried on tissue paper and frozen in liquid nitrogen.

### 2.7. Cytokine Levels

Simultaneous quantification of different cytokines in the blood serum or CSF was obtained using xMAP technology and a MAGPIX Luminex 200 platform (Thermo Fisher Scientific, Waltham, MA, USA), as recently described in detail [6]. The results were analyzed with xPONENT 4.2^®^ software (Luminex, Austin, TX, USA) and expressed as pg/mL.

### 2.8. Anti-IL6 and Anti-IL6R Monoclonal Antibodies

Rat anti-mouse-IL-6 IgG1 monoclonal antibody (mAb) (BioXCell, BE0046, Lebanon, NH, USA, RRID AB1107709) or rat anti-mouse-IL-6R IgG2 mAb (BioXCell, BE0047, RRID AB1107588) was administered at a dose of 400 μg (i.p.) per mouse daily, based on previously reported in vivo effective doses [22,23].

### 2.9. Isolation, Culture and Perifusion of Astrocytes

Isolation and culture of astrocytes from mouse adult spinal cords was based on the methodology described by Beaudet et al. [24]. A perifusion system was designed for cultured astrocytes, where a modified cultured flask was designed to have an inflow tube placed across the flask screw cap and an outflow tube placed on the flask wall opposite the screw cap. The system included a reservoir for the culture medium (including a gas diffuser to ensure its saturation using a mixture of O_2_/CO_2_ (19:1)), a roller pump, a bubble trap and a filter (Amicon YM30, Bedford, MA, USA) placed at the screw cap. The culture flask was partially submerged in a thermostatized water bath at a constant temperature of 37 °C. Effluent flow was monitored continuously for O_2_ and pH with Philips electrodes. The perifusion flow rate was of 2 mL/min.

### 2.10. H_2_O_2_ and O_2_^•^^−^ Generation Assays

Determination of H_2_O_2_ generation was based on H_2_O_2_-dependent oxidation of homovanillic acid (4-hydroxy-3-methoxyphenylacetic acid) to a highly fluorescent dimer (2,2′-dihydroxydiphenyl-5,5′-diacetic acid) mediated by horseradish peroxidase [25].

O_2_^•^^−^ was quantitated using an electrochemical biosensor, as previously described [26]. To this end, a Co_3_(PO_4_)_2_ nanorod/glassy carbon electrode was applied in situ to electrochemically detect O_2_^•^^−^ released from cells (5 × 10^5^/mL) in real time. To ensure accuracy of measured O_2_^•^^−^ concentrations, cell culture medium inside the device was mildly stirred during measurement of cell-released O_2_^•^^−^. A CHI760 electrochemical workstation (CH Instruments Inc., Austin, TX, USA) was used for electrochemical measurements.

### 2.11. Analysis of Amino Acids

Arterial blood was collected from the left ventricle into a heparinized syringe/tube. After centrifugation (10 min, 800× *g*), plasma was collected, and protein was precipitated by mixing 1 mL of plasma and 4 mL of 3.75% (*w*/*v*) sulphosalicylic acid in 0.3 M-lithium citrate buffer (pH 2.8). The supernatant (0.5 mL) was injected into an LA8080 high-speed amino-acid analyzer (Hitachi, Tokyo, Japan) for determination of amino acids.

### 2.12. RT-PCR

RNA was extracted using Qiagen RNAeasy mini kits (Hilden, Germany). Quantitative and qualitative analyses of RNA samples were performed using a 2100 Bioanalyzer (Agilent Technologies, Santa Clara, CA, USA). cDNA was obtained using a random hexamer primer and a MultiScribe reverse transcriptase kit as recommended by the manufacturer (Taq-Man RT Reagents; Thermo Fisher Scientific). PCR master mix and AmpliTaq Gold DNA polymerase were added to the specific primers (Sigma Genosys, Haverhill, UK), as previously reported [19], for GCL (γ-glutamylcysteine ligase, catalytic subunit), GGT (γ-glutamyl transpeptidase), SOD (superoxide dismutases) 1 and 2, CAT (catalase), GPX2 (glutathione peroxidase 2), GSR (glutathione reductase) and G6PDH (glucose-6-P dehydrogenase). For real-time quantification of mRNA relative to GAPDH, a SYBR Green I assay and an iCycler detection system (Biorad, Hercules, CA, USA) were used. Relative gene expression is expressed as fold change. The threshold cycle (CT) was determined, and the relative gene expression was expressed as fold change = 2^−Δ(ΔCT)^, where ΔC_T_ = C_T_ target − C_T_ GAPDH and Δ(ΔC_T_) = ΔC_T_ treated − ΔC_T_ control.

### 2.13. Enzyme Activities

GCL activity was determined by measuring the rate of ADP formation at 37 °C in a medium containing Tris/HCl buffer (pH 8.2), KCl, ATP, phosphoenolpyruvate, L-Glu, L-α-aminobutyrate, MgCl2, EDTA, NADH, pyruvate kinase and lactate dehydrogenase. GGT activity was measured using glycylglycine as a γ-glutamyl acceptor substrate and γ -glutamyl-4-nitroanilide and its carboxy derivative, γ-glutamyl-3-carboxy-4-nitroanilide, as donor substrates. SOD activity was measured using cyanide in the assay medium to distinguish the mangano-type enzyme (SOD2) from the cuprozinc type (SOD1). To measure CAT, activity samples were incubated in the presence of an H_2_O_2_ solution for 2 min prior to rapid mixing of the incubation enzymatic reaction mixture with a cobalt-bicarbonate reagent, which assesses non-reacting H_2_O_2_. Catalase activity is always directly proportional to the rate of dissociation of H_2_O_2_. GPX (selenium-dependent) activity was measured using H_2_O_2_ as a substrate in Tris-HCl buffer with the addition of NaN_3_ and EDTA (pH 8.5) at an incubation temperature of 37 °C. GSR activity follows the reduction of GSSG to GSH by monitoring the oxidation of NADPH monitored by a decrease in absorbance at 340 nm (see [25] and references therein).

### 2.14. Oxygen Consumption

O_2_ concentration and rate of O_2_ consumption in isolated astrocytes was continuously recorded using a high-resolution oxygraph (OROBOROS INSTRUMENTS Corp., Innsbruck, Austria). Isolated astrocytes were resuspended in respiration medium (DMEM 4500 mg/L glucose to which was added 5 mM pyruvate) that had been pre-warmed to 37 °C. A volume of 2.0 mL of cell suspension was added to the O_2_ electrode chamber, where it was magnetically stirred and kept at 37 °C. The chamber was sealed, and the cells were incubated until a stable respiratory rate was reached.

### 2.15. Western Blotting

Western blots were run, as previously described [25]. Proteins were transferred to a nitrocellulose membrane and subjected to Western blotting with specific anti-human monoclonal antibodies (OriGene, Rockville, MD, USA; and Abcam, Cambridge, UK). Blots were developed using horseradish-peroxidase-conjugated secondary antibody and enhanced chemiluminescence (ECL system; GE HealthCare Life Sciences, Piscataway, NJ, USA). Protein bands were quantified using laser densitometry.

### 2.16. Expression of Results and Statistical Analyses

Data are presented as the means ± SD for the indicated number of different experiments. Statistical analyses were performed using Student’s *t* test, and *p* values of <0.05 were considered significant. Survival data were analyzed with Kaplan–Meier curves and LogRank (Mantel-Cox) tests.

## 3. Results

### 3.1. Neuromotor Evaluation of ALS Mouse Models

**Figure 1:**
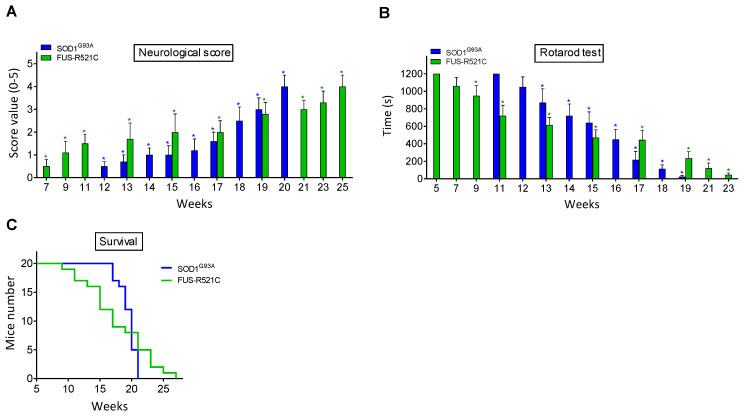
Functional performance and neuromotor coordination in SOD1^G93A^ and FUS-R521C mice. Neurological score (**A**), rotarod test (**B**) and survival (**C**) were assessed in SOD1^G93A^Q and FUS-R521C mice at different times in the progression of the disease. Taking into account the % of animals that died before reaching the advanced progression status, to assess the neurological score and the rotarod test, their number was increased so that n (20 animals) was the same both at onset and at advanced progression. In wild-type controls (*n* = 20) the neurological score was equal to 0 and the time for the rotarod test was 1200 s in all cases. Both tests were performed twice a week. On the days of the week on which the animals were not subjected to tests, they were trained in the rotarod for 20 min. Control experiments performed in wild-type mice (*n* = 10) rendered a neurological score of 0 and a performance time of 1200 s in the rotarod test in all cases. * *p* < 0.01 comparing values obtained in amyotrophic lateral sclerosis (ALS) mice versus wild-type controls.

Neuromotor functions were studied in SOD1^G93A^ and FUS-R521C mice using a standardized neurological score (designed to assess hind-limb function) (Figure 1A) and the rotarod performance test (a measure of balance, coordination, physical condition and motor planning) (Figure 1B). As shown in Figure 1, the onset of the symptomatology is around postnatal week 12 in SOD1^G93A^ mice and postnatal week 7 in the FUS-R521C model. An advanced state of disease progression was well established around postnatal weeks 17–18 (SOD1^G93A^) and 17–19 (FUS-R521C). The survival rate for both models is displayed in Figure 1C. Based on these data, further experiments were performed comparing measurements at the onset of the symptomatology and at an advanced state of progression.

### 3.2. GSH Synthesis and Release in Hepatocytes from ALS Mice

Under physiological conditions, the liver is the main reservoir and source of circulating GSH [27]. We studied the rates of GSH synthesis and efflux in isolated hepatocytes from wild-type (WT), SOD1^G93A^ and FUS-R521C mice. As shown in Table 1, in the presence of amino-acid precursors, GSH synthesis and efflux significantly increase in both models at an advanced state of disease progression, thus suggesting that an ALS-induced signaling mechanism likely promotes those changes.

We also measured liver and blood GSH levels. As shown in Table 2 and as compared to values obtained in the wild-type mice, liver GSH levels decrease in both ALS mouse models at an advance state of progression, a fact that correlates with a decrease in the ratio of synthesis/efflux (Table 1). However, the increase in liver GSH efflux does not cause the consequent increase in circulating GSH; in fact, GSH decreases in the circulating blood in both models (Table 2), a fact suggesting that blood GSH may be bypassed for metabolism. This experimental evidence raises the question of how and where GSH flow is directed during ALS progression.

### 3.3. IL6 Induces GSH Efflux in Hepatocytes

**Figure 2:**
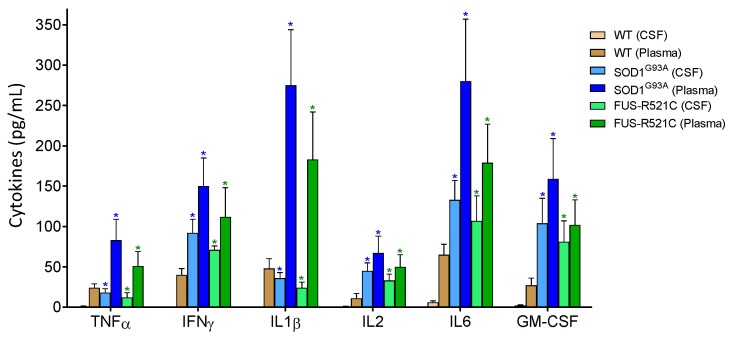
Levels of pro-inflammatory cytokines in cerebrospinal fluid (CSF) and plasma of wild-type and ALS mice. Cytokines were measured as described in Methods. To ensure that the results were comparatively homogeneous, all the mice were subjected to the tests and training indicated in Figure 1. Data in ALS mouse models correspond to an advanced state of progression. Data are mean values ± SD for *n* = 7. * *p* < 0.01 comparing values obtained in ALS models versus wild-type controls.

It is well known that ALS is associated with neuroinflammation and an increase in proinflammatory cytokines in plasma and CSF (see e.g., Obrador [6]). We measured some of these cytokines in both biological fluids in our experimental models at an advanced state of disease progression. As shown in Figure 2, as compared to healthy wild-type mice, proinflammatory cytokine levels increase. As introduced above, IL6 signaling, in particular, could play a role in increasing the release of liver GSH. We tested this hypothesis in the ALS models (at an advanced state of progression) by administering anti-IL6 mAbs or anti-IL6R mAbs in vivo. As compared to controls treated with physiological saline, both types of antibodies decreased the rate of GSH efflux from hepatocytes (Table 3), thus proving, in two different models, that IL6 is a main cytokine responsible for the increase in hepatic GSH efflux during ALS progression. In parallel experiments, we found that treatment with anti-IL6 mAbs is also associated with an increase in GSH levels in the liver (Table 3). Blood GSH levels also increased (Table 3). Therefore, either the metabolic use of circulating GSH decreases or GSH is released to the blood by other organs/tissues as a consequence of anti-IL6 treatment. In order to answer this paradox, we looked at GSH levels, GCL activity and Cys/cystine uptake in the kidneys and lungs (two organs with a significant content of GSH). As shown in Table 4, anti-IL6R treatment induces a significant decrease in GSH in both organs (suggesting that more GSH is released to the blood) and an increase in GCL activity and Cys/cystine uptake (which implies a higher level of GSH synthesis). These results suggest a systemic adaptation to the treatment (involving kidneys and lungs, although it could involve more organs). This is an adaptation that would explain the increase observed in blood GSH (Table 3).

**Figure 3:**
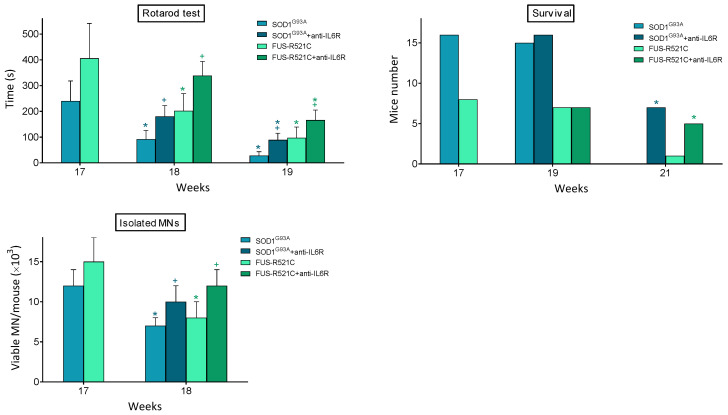
Effect of in vivo treatment with anti-IL6 mAbs on neuromotor function, survival, and MN viability of ALS mouse models. Antibody administration (one dose per day from week 17 to week 19), rotarod test (as in Figure 1), and isolation of MN were performed, as described in Methods. Data are mean values ± SD for n = 20. * *p* < 0.05 comparing weeks 18–21 versus week 17, ^+^ *p* < 0.05 comparing data obtained in anti-IL6R-treated mice versus controls treated with physiological saline.

In order to answer whether this treatment leads to a rescue in the progression of the disease, we looked at neuromotor function, survival and the number of viable motor neurons that could be isolated from anti-IL6R-treated mice. As shown in Figure 3, it is evident that anti-IL6R treatment significantly improves all these parameters. Nevertheless, behind this anti-IL6-dependent rescue, a decrease in the IL6-dependent proinflammatory effects is also possible.

### 3.4. GSH Synthesis in Astrocytes Is Highly Dependent on Extracellular GSH Supply

Based on the fact that astrocytes provide GSH to neurons [13], we investigated whether GSH levels in astrocytes are affected during ALS progression. For this purpose, we use a perifusion system (see Methods) to perfuse the cultured astrocytes with a constant flow of medium containing the same GSH levels measured in the blood of ALS mice (Table 5). Supply of amino-acid precursors is not limited during ALS progression (Table 6A). As shown in Table 5, GSH levels in astrocytes decrease at an advanced state of progression (approx. to 50%, as compared to the levels found in astrocytes isolated from WT mice or from ALS mice at the onset of symptomatology). Nevertheless, the decrease in GSH, paradoxically, is associated with an increase in GCL activity, the rate-limiting step in GSH synthesis (Table 5). GSSG levels also increase significantly, thus suggesting an increase in oxidative stress within astrocytes (Table 5). Since GSH synthesis is highly dependent on the availability of free Cys (the levels of which are extremely low inside and outside cells) [29], we also measured GGT activity and rates of Cys and cystine uptake. As shown in Table 6B, advanced ALS progression is associated with an increase in GGT activity, no changes in the rate of extracellular Cys uptake and a decrease in cystine uptake. Cells do not transport intact GSH from the extracellular space [30]. GGT is the only enzyme that cleaves the γ-glutamyl-cysteine peptide bond in GSH [31], thus releasing γ-Glu and cysteinylglycine, which is further cleaved into Cys and Gly by plasma-membrane-bound dipeptidases [32]. Free γ-glutamyl amino acids Cys and Gly can serve as GSH precursors [33]. Hence, our data suggest that extracellular GSH is the main source of Cys for astrocytes during advanced ALS progression. To further test this postulate, we investigated the effect on astrocyte GSH levels of specific inhibitors of GGT activity (acivicin) [34], Cys uptake by the ASC transport system for neutral amino acids [(1)-amino(1-(3,5-dichlorophenyl)-3,5-dimethyl-1H-pyrazol-4-yl)acetic acid, ACPP] [35] or cystine uptake by the Xc^-^ cystine/glutamate antiporter (sulfasalazine, SSZ) [36]. These are the main carriers of Cys and cystine in mammalian cells (see Obrador [37]). As shown in Table 7, GSH depletion in astrocytes is more profound in the presence of specific inhibitors of GGT or ASC-mediated Cys transport.

### 3.5. In Astrocytes, Advanced ALS Progression Is Associated with an Increase in GSH Efflux and ROS Generation

**Figure 4:**
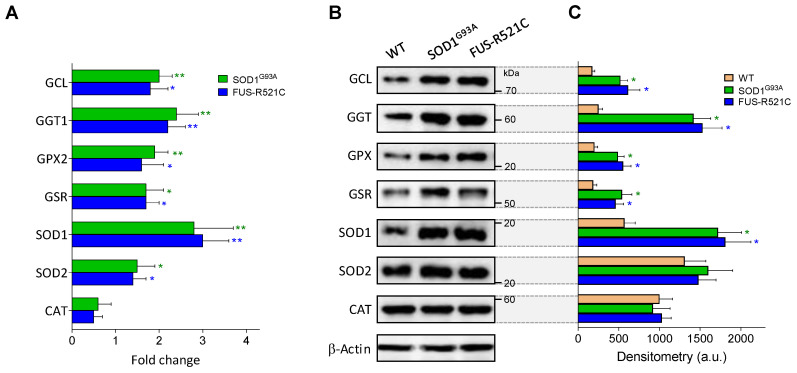
Expression and levels of GSH- and oxidative-stress-related enzymes in wild-type and ALS mice. (**A**) Data, expressed as fold change (quantitative RT-PCR; see Methods for calculations), show mean values ± SD for five different mice. (**B**) Western blots were performed in cell samples obtained from astrocytes isolated from wild-type mice and ALS mice at an advanced state of progression, thus facilitating correlations with the enzyme activities displayed in Table 5, Table 6B and Table 8. (**C**) Densitometric analysis of the Western blots represents the mean values ± SD for five different mice per molecule. * *p* < 0.05, ** *p* < 0.01 comparing ALS mice versus wild-type controls.

As described in Table 5, at an advanced state of progression, GSH levels in astrocytes decrease in the two models of ALS studied, whereas GSSG levels increase, facts suggesting an increase in oxidative stress. We further studied the underlying mechanisms, and as shown in Table 8, ROS generation increases in astrocytes isolated from ALS mice at an advanced state of progression compared to astrocytes from WT controls. The increase in ROS generation is associated with an increase in O_2_ consumption, which may reflect the high metabolic activity associated to the reactive astrocyte phenotype linked to the progression of the disease. However, in the group of enzymes studied, only SOD1 activity was found to be significantly higher in the astrocytes of the ALS mice compared to WT controls (Table 8, Figure 4). GSH efflux was also found to increase in both models, as compared to WT controls (Table 8). This increase in GSH efflux may also be facilitated by IL6 in astrocytes (as in hepatocytes), given that we observed that anti-IL6R mAbs prevents GSH depletion in perifused astrocytes (results not shown). This all indicates that the increase in ROS generation is not counteracted within the astrocytes. It is obvious that an increase in ROS generation by reactive astrocytes may cause damage to neighboring MNs. In a previous report, we observed that cytosolic GSH levels in MNs isolated from SOD1^G93A^ mice do not change as the disease progresses [6], which means that an increase in GSH efflux from astrocytes does not necessarily imply an increase in MNs.

## 4. Discussion

ALS progression is associated with severe diffuse astrogliosis, which can be a consequence of neurotoxic insults, diffuse trauma, diffuse ischemia, or different types of infection [38]. Reactive astrocytes show changes in gene expression, with pronounced upregulation of glial fibrillary acidic protein, hypertrophy and dispersed proliferation and can cause inhibition of axonal regeneration [39] and favor the arrival of inflammatory cells [40]. Therefore, mechanisms that can trigger chronic neurodegeneration. Indeed, reactive astrocytes surround degenerating MNs in ALS patients and ALS rodent models [41]. Nevertheless, the mechanisms underlying the potential damaging role of astrocytes still are a question of debate.

IL6 is upregulated in the CNS of patients affected by neuroinflammation of different etiologies [8], as well as in murine models of brain injury [42]. Its pro-inflammatory trans-signaling pathway, shown to mediate neurodegeneration in mice, depends on a soluble form of IL-6R capable of binding IL-6 and stimulating a response on distal cells that express β-receptor glycoprotein 130 [43]. IL6 knockout mice show a lower inflammatory response and neuroglial activation [44,45], increased oxidative stress [46], decreased lymphocyte recruitment [47] and a low rate of recovery and healing [48], all suggesting that IL6 may be a double-edged sword, particularly in chronic pathological processes, as is the case with ALS. As previously suggested concerning murine models of metastatic melanoma [14], we found that IL6 also promotes the release of GSH from the liver in the two ALS models assayed (Table 3). GSH synthesis and efflux are significantly increased, as compared to WT controls, in the hepatocytes of ALS mice at an advanced state of disease progression (Table 1 and Table 3). The consequent increase in blood GSH facilitates an interorgan/cellular flow that can be potentially used by astrocytes and transferred to MNs. Overall, this mechanism represents a protective effect for MNs, promoted by the increased levels of IL6 in blood and CSF (Figure 2).

Binding of IL6 to its receptor initiates a signal-transduction cascade through different transcription factors, Janus kinases (JAKs) and signal transducers, as well as activators of transcription (STATs) [49]. Whether treatment with anti-IL6 or anti-IL6R alter the expression of the mutant SOD1 and/or the mutant FUS is unknown and should be investigated. What is remarkable is the fact that despite the different phenotypes of the two ALS models, the effect of anti-IL6 treatment on GSH levels is quite similar (Table 3).

It has been demonstrated that astrocytes provide Cys to neurons by releasing GSH [13] via the multi-drug-resistant protein 1 [50]. Astrocytes can degrade extracellular GSH via GGT and release Cys-Gly, which is broken down by the action of a plasma-membrane-bound dipeptidase [37]. Then, free Cys, which is rate-limiting for GSH synthesis, can be taken up through the ASC system [37] (Table 7). The blood supply of all other amino-acid precursors for GSH synthesis is not limited in ALS mice (Table 6A). Importantly, although the rate of cystine uptake by astrocytes decreases as the disease progresses, the rate of Cys uptake is not affected (Table 6B). The effect of the inhibitors (Table 7) seems to be relative to the initial control GSH levels in each case. What is important is the fact that inhibition of GGT activity or Cys uptake causes a higher GSH depletion than inhibition of cystine uptake. Cys levels are extremely low (outside and inside cells), and consequently, Cys uptake is highly dependent on the generation of Cys from extracellular GSH (via the reaction catalyzed by GGT). GGT activity in astrocytes was found to increase, as compared to WT controls, in ALS mice (Table 6B). However, GGT activity in MNs isolated from SOD1^G93A^ mice at an advanced state of progression is lower than its equivalent in astrocytes [6]. Since astrocytes outnumber neurons by over fivefold [51], it is obvious that astrocytes have the highest capacity to metabolize extracellular GSH. Taken together, these experimental facts indicate that GSH synthesis in MNs is highly dependent on GSH supply from astrocytes, which, in turn, depends on the interorgan flow from the liver controlled by IL6.

As shown in Table 8, in both ALS models, generation of ROS by astrocytes increases at an advanced state of disease progression. Previously, we found that GSH levels in MNs isolated from SOD1^G93A^ mice at an advanced state of progression are slightly higher than the levels measured in MNs from WT control mice [6]. However, GSSG levels (which indirectly reflect a higher exposure to ROS) increase by approx. 100% [6]. An increase in O_2_ consumption and GSH efflux (Table 8), as well as the consequent decrease in GSH levels (Table 5), may explain the increase in ROS generation by astrocytes. ROS could be a direct cause of oxidative damage in neighboring MNs. The fact that SOD1 and SOD2, two key antioxidant enzyme activities, decrease in MNs of SOD1^G93A^ mice as the disease progresses [6] sets up a cumulative-damage scenario. As shown in Table 5, GSH levels decrease in astrocytes isolated from ALS mice at an advanced state of progression, compared to onset or WT. However, GCL activity increases (Table 5), and Cys uptake remains unchanged (Table 6B). Nevertheless, the increases in GSSG (Table 5) and ROS generation (both indicating more oxidative stress), as well as the increase in GSH efflux (Table 8), explain why GSH decreases. An increase in ROS exposure and a decrease in its antioxidant-defense capacity may be key factors in the death of MNs.

## 5. Conclusions

An increase in circulating levels of IL6 (released by glial and inflammatory cells) promotes the release of GSH from the liver (a physiological reservoir of this tripeptide). The increased availability of extracellular GSH facilitates its transfer, via astrocytes, to MNs and favor its antioxidant defense. However, in advanced stages of the disease, this process is associated with an increase in ROS generation by astrocytes. Oxidative stress can cause cumulative damage to MNs, as well as their death. From the results described herein, it seems obvious that maintaining GSH levels in astrocytes, and consequently in MNs, can help to slow down the progression of the disease. Since the availability of Cys is a limiting factor for the synthesis of GSH, a direct donor of Cys, e.g., N-acetylcysteine, which easily crosses the plasma membrane, could be a direct drug solution. Nevertheless, maintenance of GSH homeostasis in ALS is only part of the potential benefits of combined strategies targeting different pathways/molecules at the same time [52].

## Figures and Tables

**Table 1:** Glutathione (GSH) synthesis and efflux in isolated hepatocytes from wild-type and ALS mice. The initial GSH concentration was 5.2 ± 0.3 μmol/g in isolated hepatocytes from wild-type mice, 5.0 ± 0.4 and 4.0 ± 0.3 μmol/g in isolated hepatocytes from SOD1^G93A^ mice (at onset and advanced state of progression, respectively) and 4.8 ± 0.4 and 3.7 ± 0.3 μmol/g in isolated hepatocytes from FUS-R521C mice (at onset and advanced state of progression, respectively). Hepatocytes were incubated in 10-mL Erlenmeyer flasks (final volume 2 mL) for 60 min (see Methods) in the presence or absence of amino-acid precursors for GSH synthesis (5 mM Gln, 2 mM Gly, 1 mM Ser, 1 mM N-acetylcysteine) [28]. Only L-amino acids were used. Glucose (5 mM) and bovine serum albumin (2%) were present in all incubations. Rates of GSH synthesis were calculated from total GSH content in incubations at 0, 20, 40 and 60 min. Rates of glutathione efflux were calculated from contents of GSH and glutathione disulfide (GSSG) in the extracellular medium at 0, 20, 40 and 60 min. All values are means ± SD for 7–8 observations. For both groups, the significance test refers to the comparison of rates in hepatocytes isolated from ALS mice versus those from the wild-type controls (* *p* < 0.05, ** *p* < 0.01).

	GSH Synthesis (nmol/g·min)					GSH Efflux (nmol/g·min)				
	WT	SOD1^G93A^		FUS-R521C		WT	SOD1^G93A^		FUS-R521C	
Additions		Onset	Adv.	Onset	Adv.		Onset	Adv.	Onset	Adv.
None	2 ± 0.3	3 ± 0.4 *	5 ± 1 **	2 ± 0.5	4 ± 1 **	2 ± 0.5	4 ± 0.7 **	5 ± 2 **	3 ± 1	4 ± 1 *
L-Amino acids	22 ± 5	24 ± 4	31 ± 4 *	23 ± 3	30 ± 4 *	9 ± 3	11 ± 3	21 ± 4 **	10 ± 2	20 ± 3 **

**Table 2:** Blood and liver GSH in wild-type and ALS mice. Blood and liver samples were obtained and treated as described in Methods. Data are means ± SD for 10–12 different mice. * *p* < 0.01 comparing values obtained in ALS mice versus wild-type controls.

	WT	SOD1^G93A^		FUS-R521C	
		Onset	Adv.	Onset	Adv.
Blood GSH (μmol/gHb)	7.2 ± 0.5	7.0 ± 0.4	4.5 ± 0.4 *	7.3 ± 0.6	5.0 ± 0.5 *
Liver GSH (μmol/g of tissue)	6.9 ± 0.6	6.0 ± 0.5	3.5 ± 0.4 *	6.4 ± 0.5	3.9 ± 0.3 *

**Table 3:** Administration of anti-IL6 or anti-IL6R mAbs decreases GSH efflux from hepatocytes. Antibodies were administered, as described in Methods, during the three days prior to isolation of hepatocytes or collection of blood or liver samples. See also the captions of Table 1; Table 2. Rates of GSH efflux were determined in hepatocytes incubated in the presence of amino-acid precursors (as in Table 1). Data in ALS mouse models correspond to an advanced state of progression. Data are mean values ± SD for *n* = 9–10. * *p* < 0.01 comparing values obtained in ALS models versus wild-type controls. ^+^ *p* < 0.01 values obtained in mice treated with anti-IL6 or anti-IL6R mAbs versus controls treated with physiological saline.

	GSH Efflux from Hepatocytes (nmol/g·min)			Blood GSH (μmol/gHb)			Liver GSH (μmol/g of Tissue)		
In Vivo Administration	WT	SOD1^G93A^	FUS-R521C	WT	SOD1^G93A^	FUS-R521C	WT	SOD1^G93A^	FUS-R521C
Physiological saline	10 ± 4	20 ± 3 *	18 ± 3 *	7.4 ± 0.6	4.5 ± 0.5 *	5.2 ± 1.0 *	7.1 ± 0.5	3.6 ± 0.7 *	3.8 ± 0.7 *
Anti-IL6	8 ± 3	12 ± 3 ^+^	11 ± 2 ^+^	7.9 ± 0.5	7.0 ± 0.8 ^+^	7.2 ± 0.8 ^+^	7.5 ± 0.9	6.5 ± 0.5 ^+^	6.9 ± 1.0 ^+^
Anti-IL6R	8 ± 2	10 ± 2 ^+^	10 ± 3 ^+^	8.0 ± 0.9	7.4 ± 0.7 ^+^	7.5 ± 0.6 ^+^	7.6 ± 1.0	6.9 ± 0.8 ^+^	7.1 ± 0.5 ^+^

**Table 4:** Effect of in vivo treatment with anti-IL6 mAbs on GSH levels, GCL activity and Cys/cystine uptake in kidneys and lungs of the ALS mouse models. Antibodies were administered, as described in Methods, during the seven days prior to assay of all parameters. Rates of Cys and cystine were calculated after administering i.v. 2.0 μCi of [^35^S]Cys or 10.0 μCi of [^35^S]cystine (PerkinElmer, Waltham, MA, USA). Data are mean values ± SD for *n* = 5–6. * *p* < 0.05, ** *p* < 0.01 comparing data for each parameter obtained in anti-IL6R-treated mice versus controls treated with physiological saline.

	SOD1^G93A^				FUS-R521C			
	Kidneys		Lungs		Kidneys		Lungs	
Anti-IL6R	−	+	−	+	−	+	−	+
GSH (μmol/g of tissue)	3.2 ± 0.4	1.4 ± 0.4 **	1.7 ± 0.3	0.9 ± 0.3 **	3.0 ± 0.5	1.2 ± 0.4 **	1.5 ± 0.3	0.8 ± 0.2 **
GCL (U/g of tissue)	92 ± 17	155 ± 26 **	49 ± 15	96 ± 24 *	85 ± 21	148 ± 36 *	41 ± 8	88 ± 12 **
Cys uptake (nmol/mg prot·min)	1.2 ± 0.3	2.5 ± 0.8 *	0.7 ± 0.1	1.6 ± 0.4 **	1.0 ± 0.2	2.0 ± 0.4 **	0.6 ± 0.1	1.2 ± 0.3 **
Cystine uptake (nmol/mg prot·min)	11.5 ± 1.5	16.9 ± 2.7 *	5.4 ± 1.0	10.3 ± 1.7 **	13.1 ± 1.7	19.5 ± 2.6 **	5.9 ± 0.9	9.4 ± 1.8 **

**Table 5:** GSH/GSSG levels and GCL activity in astrocytes isolated from wild-type and ALS mice. Data are mean values ± SD for *n* = 9. * *p* < 0.01 comparing values obtained in ALS models versus the wild-type controls. ^+^ *p* < 0.01 comparing advanced state of progression versus onset of symptomatology.

	WT	SOD1^G93A^		FUS-R521C	
		Onset	Adv.	Onset	Adv.
GSH (nmol/10^6^ cells)	23.1 ± 3.1	24.5 ± 2.7	12.9 ± 1.8 *^,+^	22.3 ± 2.5	13.7 ± 2.9 *^,+^
GSSG (nmol/10^6^ cells)	0.7 ± 0.2	0.8 ± 0.2	1.9 ± 0.4 *^,+^	0.7 ± 0.3	1.6 ± 0.5 *^,+^
GCL (mU/10^6^ cells)	68 ± 17	75 ± 14	123 ± 25 *^,+^	65 ± 12	117 ± 31 *^,+^

**Table 6:** Supply of amino-acid precursors for GSH synthesis, GGT activity and cyst(e)ine uptake in astrocytes isolated from wild-type and ALS mice at an advanced state of progression. (**A**) Amino-acid levels in circulating blood. (**B**) GGT activity and rates of Cys and cystine uptake. To measure Cys and cystine uptake, astrocytes were incubated in the presence of 0.2 μCi/mL of [^35^S]Cys and 10 uM Cys or 0.5 μCi/mL of [^35^S]cystine and 100 uM cystine (labeled amino acids were obtained from PerkinElmer, Waltham, MA, USA). Maximum rates of Cys and cystine uptake were reached at approx. 2 and 20 min of incubation, respectively. Data are mean values ± SD for *n* = 7. * *p* < 0.01 comparing values obtained in ALS models versus wild-type controls.

A	Amino Acid Concentration (μM) in Whole Blood		
	WT	SOD1^G93A^	FUS-R521C
Gln	510 ± 63	493 ± 29	455 ± 55
Glu	123 ± 36	136 ± 21	115 ± 29
Gly	306 ± 77	277 ± 45	285 ± 53
Ser	151 ± 27	140 ± 31	130 ± 20
Meth	55 ± 12	50 ± 10	46 ± 9
Cyst(e)ine	18 ± 3	14 ± 2	15 ± 3
**B**	**GGT Activity and Cyst(e)ine Uptake**		
	**WT**	**SOD1^G93A^**	**FUS-R521C**
GGT (mU/10^6^ cells)	15.3 ± 2.4	25.7 ± 3.9 *	23.2 ± 4.0 *
Cys uptake (nmol/mg prot·min)	2.1 ± 0.5	2.0 ± 0.3	2.2 ± 0.3
Cystine uptake (nmol/mg prot·min)	24.5 ± 3.5	10.6 ± 1.7 *	11.7 ± 2.2 *

**Table 7:** Effect of inhibition of GGT activity or Cys/cystine uptake on GSH levels in astrocytes. Astrocytes isolated from wild-type and ALS mice at an advanced state of progression were incubated in the absence or presence of acivicin (0.05 mM), ACPP [(1)-amino(1-(3,5-dichlorophenyl)-3,5-dimethyl-1H-pyrazol-4-yl)acetic acid, 0.1 mM] or SSZ (sulfasalazine, 0.2 mM) during a 24 h-time period before measuring GSH levels. The culture medium contained extracellular GSH and cyst(e)ine at a concentration of 10 a 1 mM, respectively. Data are mean values ± SD for *n* = 5–6. * *p* < 0.01 comparing values obtained in ALS models versus wild-type controls. ^+^ *p* < 0.01 comparing the different inhibitors versus controls.

	GSH (nmol/10^6^ Cells)		
	WT	SOD1^G93A^	FUS-R521C
Control	22.7 ± 3.4	13.2 ± 2.0 *	14.1 ± 2.4 *
Acivicin	7.5 ± 1.7 ^+^	3.1 ± 1.1 *^,+^	3.0 ± 0.7 *^,+^
ACPP	8.0 ± 1.9 ^+^	3.5 ± 1.2 *^,+^	3.1 ± 0.9 *^,+^
SSZ	16.6 ± 2.3 ^+^	10.2 ± 1.8 *	11.3 ± 1.5 *

**Table 8:** ROS generation and GSH efflux in astrocytes isolated from wild-type and ALS mice. All parameters (see Methods) were determined in astrocytes isolated from wild-type or ALS mice at an advanced state of progression. Data are mean values ± SD for *n* = 9. * *p* < 0.01 comparing values obtained in ALS models versus wild-type controls.

	WT	SOD1^G93A^	FUS-R521C
**ROS**			
H_2_O_2_ (nmol/10^6^ cells·min)	0.69 ± 0.08	1.38 ± 0.29 *	1.16 ± 0.24 *
O_2_^●−^ (nmol/10^6^ cells·min)	0.30 ± 0.05	0.77 ± 0.12 *	0.63 ± 0.17 *
ROS and GSH metabolism			
SOD1 (U/10^6^ cells)	0.81 ± 0.14	1.62 ± 0.15 *	1.74 ± 0.28 *
SOD2 (U/10^6^ cells)	0.23 ± 0.05	0.31 ± 0.07	0.29 ± 0.05
CAT (mU/10^6^ cells)	1.50 ± 0.24	1.85 ± 0.36	2.14 ± 0.51
GPX (mU/10^6^ cells)	3.06 ± 0.84	4.25 ± 0.96	5.06 ± 0.71 *
GSR (mU/10^6^ cells)	5.22 ± 0.53	7.70 ± 1.25 *	7.20 ± 1.46
GSH efflux (nmol/10^6^ cells·h)	5.3 ± 0.8	9.4 ± 1.3 *	10.2 ± 1.5 *
O_2_ consumption (pmol/10^6^ cells·min)	483 ± 63	725 ± 84 *	667 ± 75 *

## Data Availability

Data are contained within the article.

## Abbreviations

ALS, amyotrophic lateral sclerosis; GSH, glutathione; CNS, central nervous system; MN, motor neuron; SALS, sporadic ALS, FALS, familial ALS; IL6, interleukin 6; IL6R, IL6 receptor; ROS, reactive oxygen species; GSSG, glutathione disulfide; GCL, γ-glutamyl-cysteinyl ligase; GGT, γ-glutamyl transpeptidase; SOD, superoxide dismutase; CAT, catalase, GPX, glutathione peroxidase; GSR, glutathione reductase; G6PDH, glucose-6-P dehydrogenase; WT, wild type; mAbs, monoclonal antibodies.
